# Noninvasive assessment of characteristics of novel anti-HER2 antibodies by molecular imaging in a human gastric cancer xenograft-bearing mouse model

**DOI:** 10.1038/s41598-018-32094-x

**Published:** 2018-09-13

**Authors:** Wei-Ying Kuo, Jia-Jia Lin, Hung-Ju Hsu, Hong-Sen Chen, An-Suei Yang, Chun-Yi Wu

**Affiliations:** 1 Genomics Research Center, Academia Sinica, Taipei, Taiwan; 2 Department of Biomedical Imaging and Radiological Science, China Medical University, Taichung, Taiwan; 3 Master Program for Biomedical Engineering, China Medical University, Taichung, Taiwan

## Abstract

Human epidermal growth factor receptor 2 (HER2) overexpression occurs in various types of cancers. Regarding the anti-HER2 targeted therapies showed superior treatment outcomes in several (pre)clinical studies, we used multimodality image to rapidly select novel HER2-targeting antibodies for further therapeutics development. The four anti-HER2 antibodies (H32 IgG, 75 IgG, 61 IgG, and trastuzumab) labeled with either In-111 or a DyLight680 fluorescent dye were applied to perform cellular uptake, endocytosis, optical/microSPECT/CT imaging and biodistribution studies. *In vitro* and *in vivo* relative effectiveness of these antibodies were also compared in an N87 gastric cancer xenograft model. The internalized radioactivity of [^111^In]61 IgG in N87 cells increased from 33% at 12 hr to 56% at 48 hr after incubation, while the majority of other antibodies stayed on the cell membranes. Among these antibodies, 61 IgG showed the highest accumulation in tumors with the tumor-to-muscle ratio (*T/M*) of 131 ± 61.4 and 19.13 ± 3.42 conducted by IVIS and microSPECT/CT, respectively. We demonstrated that multimodality imaging is a reliable approach for selecting potential antibodies and found that 61 IgG manifested significant tumor accumulation with elevated internalization rate thus could be a suitable candidate for further development of new HER2-targeted therapies.

## Introduction

Gastric cancer is the fourth most common cancer and the fourth leading cause of cancer death around the world, accounting for an estimated 28,000 new cases and 10,960 deaths in 2017^1^. Although its incidence and mortality rate declined steadily over the last half-century due to important advances in our understanding of the disease, the five-year survival rate is still 30.6% in the United States. Clinically, the symptoms of gastric cancer usually emerge late, and most patients are diagnosed at the advanced stages of the disease, which contributes to poor prognosis^2^. Therefore, development of methods for early detection and treatment for improving gastric cancer outcome is urgently warranted.

Antibodies have high specificity toward their targets, which places them an ideal imaging probe to monitor disease progression and assist medical decisions in preclinical and clinical studies. It is evident that radiolabeled antibody-targeting is a more reliable way for assessment of HER2 expression in patients than conventional immunohistochemistry (IHC) and fluorescence *in situ* hybridization (FISH)^3,4^. The main reason is that biopsied tissues merely represent a limited part of the whole tumor, hindering the careful consideration of complex intratumoral heterogeneity. Furthermore, the biopsy approach exhibits the potential risk of causing metastasis during the procedure^5^.

Trastuzumab, the first Food and Drug Administration (FDA)-approved HER2-targeting recombinant humanized monoclonal antibody for the treatment of breast cancer, has been labeled with different radioisotopes for diagnosis and radiation therapy^6–8^. A similar immunoreactivity and tumor uptake between ^89^Zr-trastuzumab and ^111^In- trastuzumab in breast cancer xenograft models was noticed^7^. In fact, antibodies binding to different epitopes of HER2 may result in a different level of affinity, accumulation and following biological signaling. In this study, we aim to utilize molecular imaging for rapid *in vivo* selection of valuable self-developed antibodies.

An antibody-drug conjugate (ADC), named trastuzumab-emtansine (T-DM1), reached the market in 2013 for the treatment of HER2-positive metastatic breast cancer and showed an impressive clinical efficacy^9,10^. Unfortunately, some patients do not respond to T-DM1 or acquire resistance and then relapse^11^. At present, ^89^Zr-trastuzumab has been used to detect HER2-positive metastatic in patients or select the cases that may benefit from T-DM1 treatment^12,13^. T-DM1 also failed Phase II/III trial against gastric cancer in 2015 (NCT01641939). The development of novel antibodies to prepare more potent anti-HER2 ADCs for cancer therapy is becoming a promising field in oncology. Marquez *et al*. showed that increased tumor uptake of ^89^Zr-pertuzumab in the presence of unlabeled trastuzumab^14^, implying its better affinity toward HER2 and the potential of using pertuzumab-derived ADC to treat patients.

To assess the effect of antibody epitope on the biologic process, a phage-displayed synthetic antibody library (GH2), designed based on computational analyses and experimental investigation, was constructed for the production of antibodies with a single human variable domain antibody germline framework in our previous study^15^. Hundreds of antibodies specifically against HER2-ECD (human epidermal growth factor receptor 2 – extracellular domain) with novel epitopes have been discovered from this library. The rate of internalization or target turnover rate is a crucial parameter that can accelerate selection for appropriate ADC candidates from a large pool of antibodies. In this study, we applied either fluorescent dye- or radioisotope-labeled antibodies to investigate their *in vivo* distributions and compare the rates of internalization among trastuzumab and artificially synthesized anti-HER2 antibodies. Combining the synthetic antibody libraries with the *in vivo* noninvasive imaging platform enables efficient selection with faster and more accurate judgments for further developing optimally functional ADCs.

## Results

### The Binding capacity of Dye-anti-HER2 antibody to HER2-positive cancer cells

**Figure 1:**
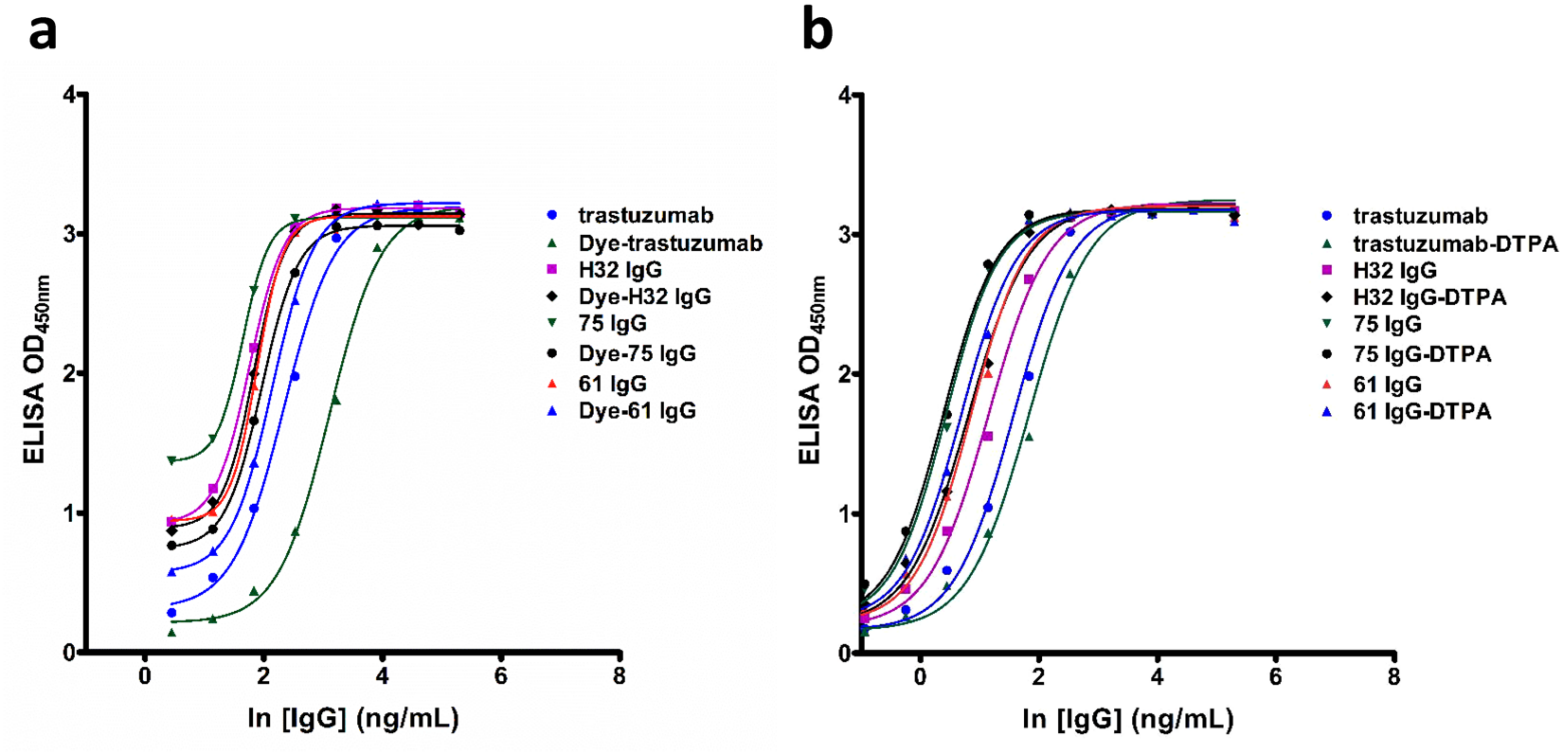
ELISA analysis of parent and (**a**) dye- and (**b**) DTPA-conjugated antibodies.

The antigen binding affinity of anti-HER2 antibodies and their derivatives to HER2 ECD was analyzed by enzyme-linked immunosorbent assay (ELISA) (Fig. 1a), demonstrating that the target affinities of our antibodies, including H32 IgG, 61 IgG, and 75IgG were all higher than that of trastuzumab. The conjugation of DyLight680 Dye to these anti-HER2 antibodies affects affinities to an extent but no more than 3-fold of affinity lost. Generally, only a moderate difference in target affinity between modified (diethylenetriaminepentaacetic acid (DTPA)-conjugated) antibodies and parent antibodies (Fig. 1b).

### *In vitro* cellular uptake and internalization assays

**Figure 2:**
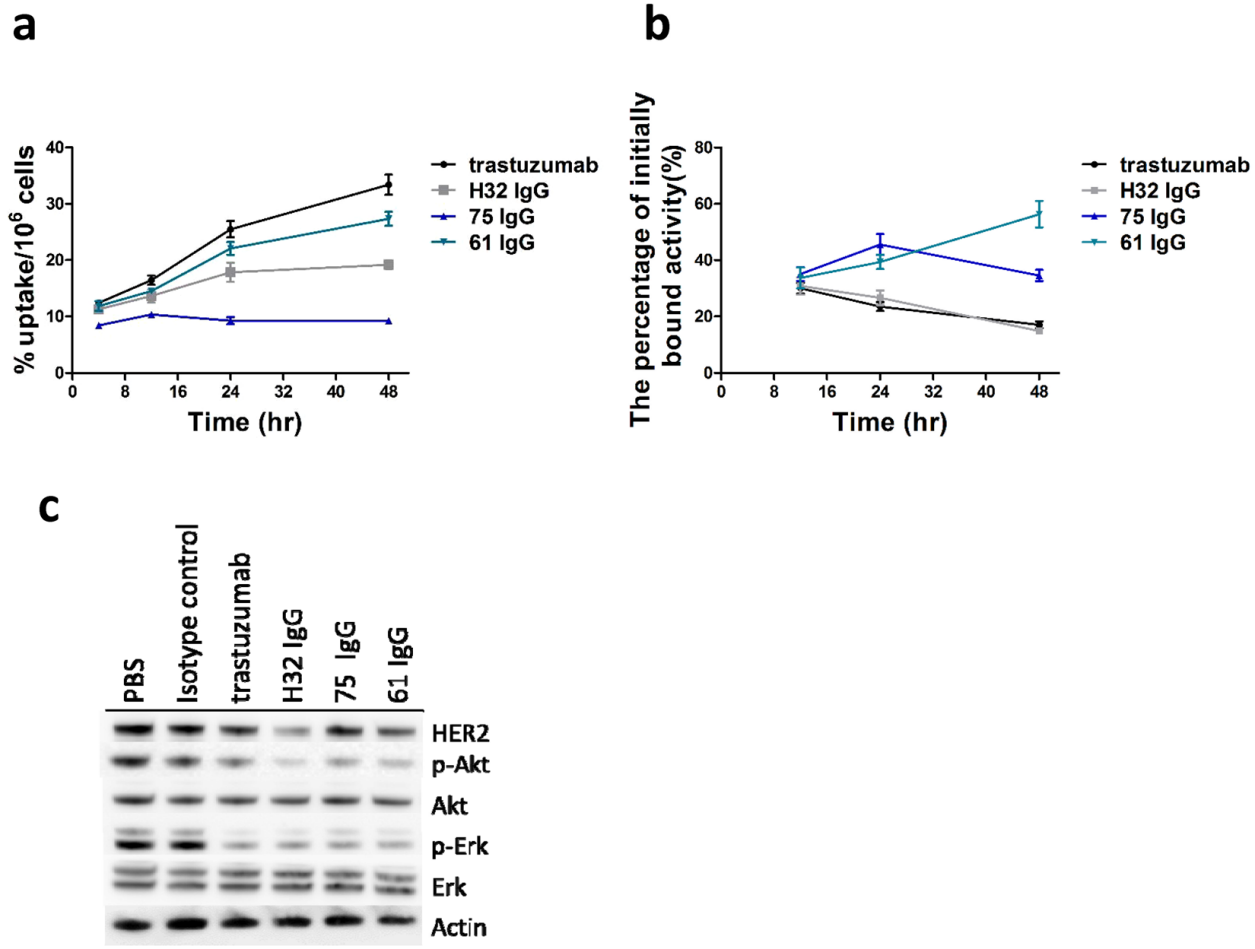
(**a**) The cellular uptake of ^111^In-labeled antibodies in N87 cells at 4, 12, 24, and 48 hr after incubation. (**b**) Internalization of ^111^In-labeled antibodies by N87 cells with respect to the total amount of cell-bound radioactivity.

The cellular uptake of [^111^In]H32 IgG, [^111^In]61 IgG, [^111^In]75 IgG and [^111^In]trastuzumab expressed as %AD/10^6^ cells, increased with time and reached the maximum accumulation of 19.18 ± 0.74, 27.34 ± 1.23, 9.27 ± 0.26, and 33.40 ± 1.77 at 48 hr post-incubation, respectively. The results indicated a specific targeting ability against HER2-overexpressing cells (Fig. 2a).

For internalization assays, the percentage of initially bound activity of these antibodies was shown in Fig. 2b. Although the cellular uptake of [^111^In]trastuzumab was highest among these antibodies, its internalized fraction decreased with time rapidly. The percentage of radioactivity of [^111^In]61 IgG internalized in cells increased from 33.67 ± 3.68 at 12 hr to 56.32 ± 4.71 at 48 hr after incubation, while that of [^111^In]75 IgG exhibited an initial increment and then dropping off.

### Western blot analyses following treatment of N87 cells with anti-HER2 antibodies

N87 cells treated with 10 μg/mL of anti-HER2 IgGs as indicated in Fig. 2c. The antibody-induced depletion of membrane HER2 was noticed in H32 and 61 IgGs treatments when compared with trastuzumab one. For inhibition of downstream signaling, the expression of phospho-AKT (p-Akt) and phospho-ERK (p-Erk) in the cells co-incubated with H32, 61, 75 IgGs as well as trastuzumab was significantly lower than the controls, suggesting the clinical potential of these self-developed antibodies.

### Effect of Dye-anti-HER2 antibodies on N87 cells

**Figure 3:**
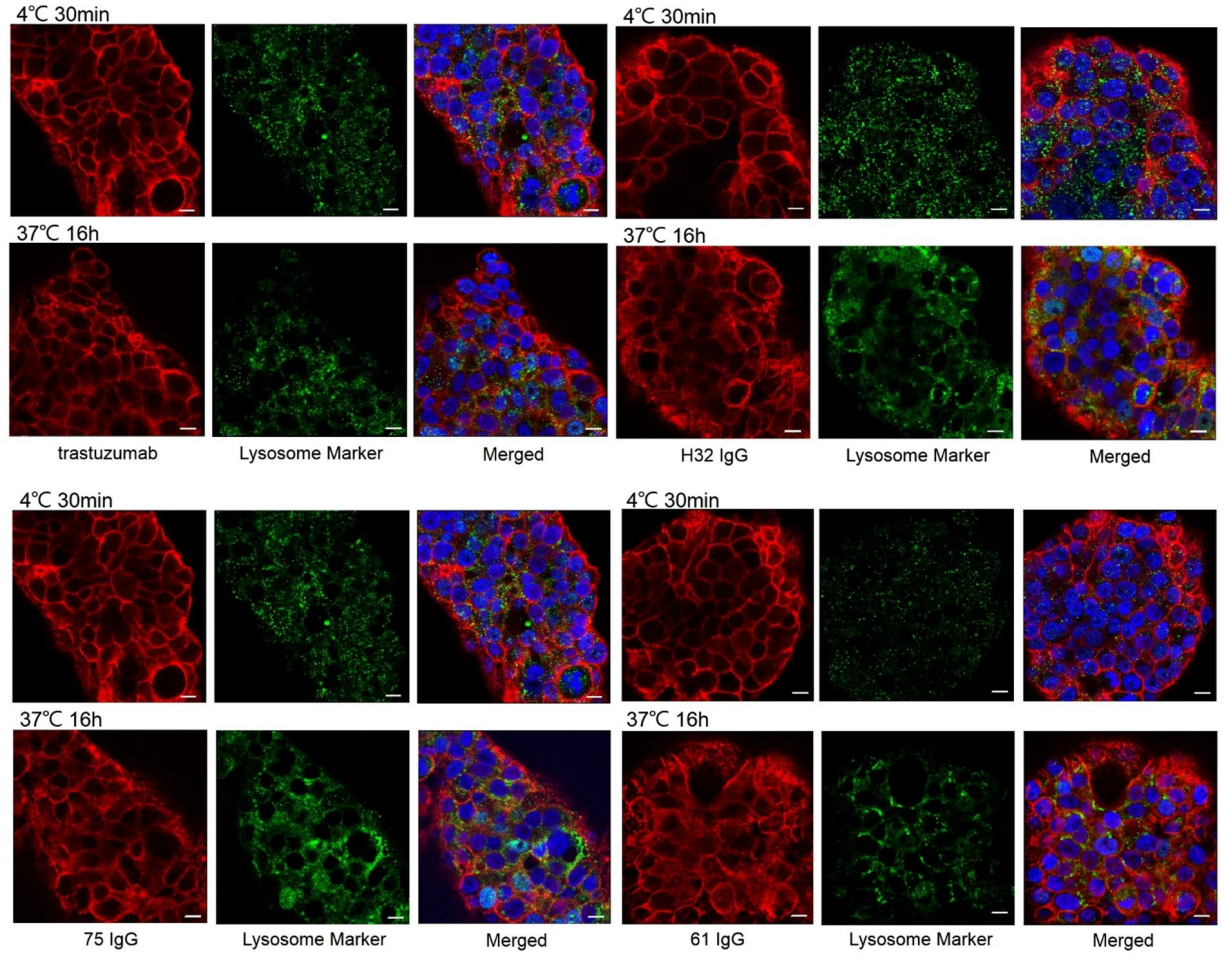
Confocal microscopy images of N87 cells incubated with dye-conjugated antibodies either at 4 °C for 30 mins or at 37 °C for 16 hr. Scale bar = 10 μm.

The levels of internalization between anti-HER2 antibodies in N87 cells were assessed by immunofluorescence staining (Fig. 3). The majority of trastuzumab and H32 IgG still stay on the cell membrane even though after the incubation at 37 °C for 16 hr. On the contrary, 61 IgG and 75 IgG initially detected on the surface of the cells at 0 °C whereas they were internalized and the signals of intracellular IgG were colocalized with lysosomes at 16 hr after incubation at 37 °C.

### Near-infrared fluorescence (NIRF) imaging of Dye-conjugated IgGs in subcutaneous xenografts

**Figure 4:**
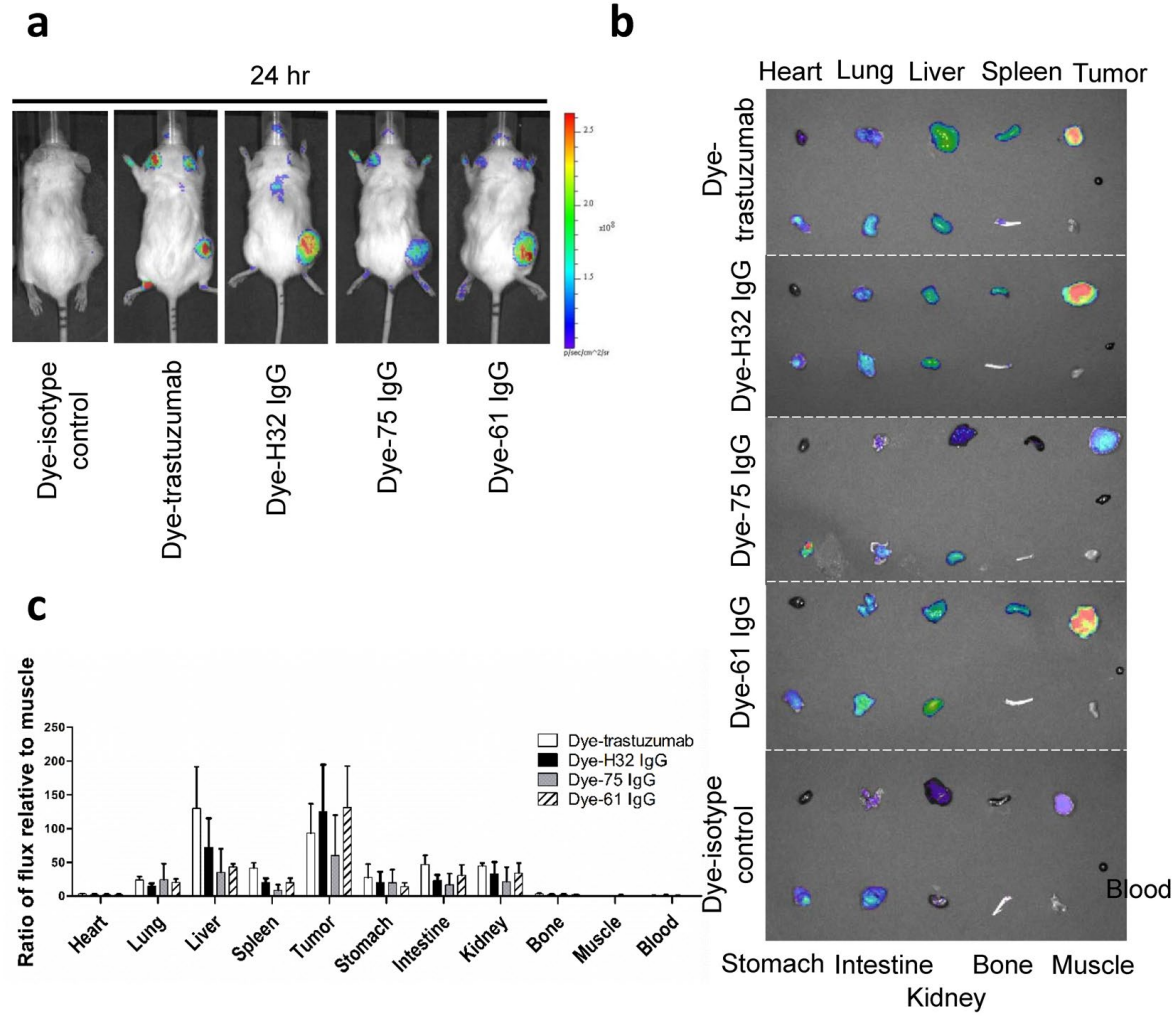
(**a**) *In vivo* NIRF imaging of N87 xenograft-bearing mice. (**b**) *Ex vivo* NIRF imaging of organs. (**c**) The quantification of *ex vivo* imaging of excised organs.

Optical scans of N87 xenograft-bearing mice injected with Dye-anti-HER2 IgGs and Dye-isotype control (negative control) at 24 hr p.i. were shown in Fig. 4a. The signals of Dye-anti-HER2 IgGs were clearly observed with high contrast relative to the background at the tumor site. However, negative control IgG showed nearly no tumor uptake due to lack of HER2-targeting ability. *Ex vivo* NIRF imaging showed that the uptake of Dye-anti-HER2 IgGs in most normal organs represented similarity to that of the negative control (Fig. 4b,c). The tumor uptake of Dye-anti-HER2 IgGs (124.7 ± 69.3 for H32 IgG; 131 ± 61.4 for 61 IgG; 71.3 ± 39.4 for 75 IgG; 92.9 ± 43.8 for trastuzumab) was significantly higher than that of the negative control (35.8 ± 2.1, P < 0.01). The results were consistent with the findings obtained from *in vivo* NIRF imaging. We observed that Dye-61 IgG exhibited higher tumor uptake and lower liver uptake than that of Dye-trastuzumab (P < 0.05). The similar results were observed in a BT474 human breast cancer xenograft-bearing mouse model (Figure S1a). The Dye-61 IgG also had the highest tumor accumulation when compared with Dye-H32 IgG and Dye-trastuzumab (P < 0.05).

### MicroSPECT/CT imaging of 111In-labeled IgGs in subcutaneous xenografts

**Figure 5:**
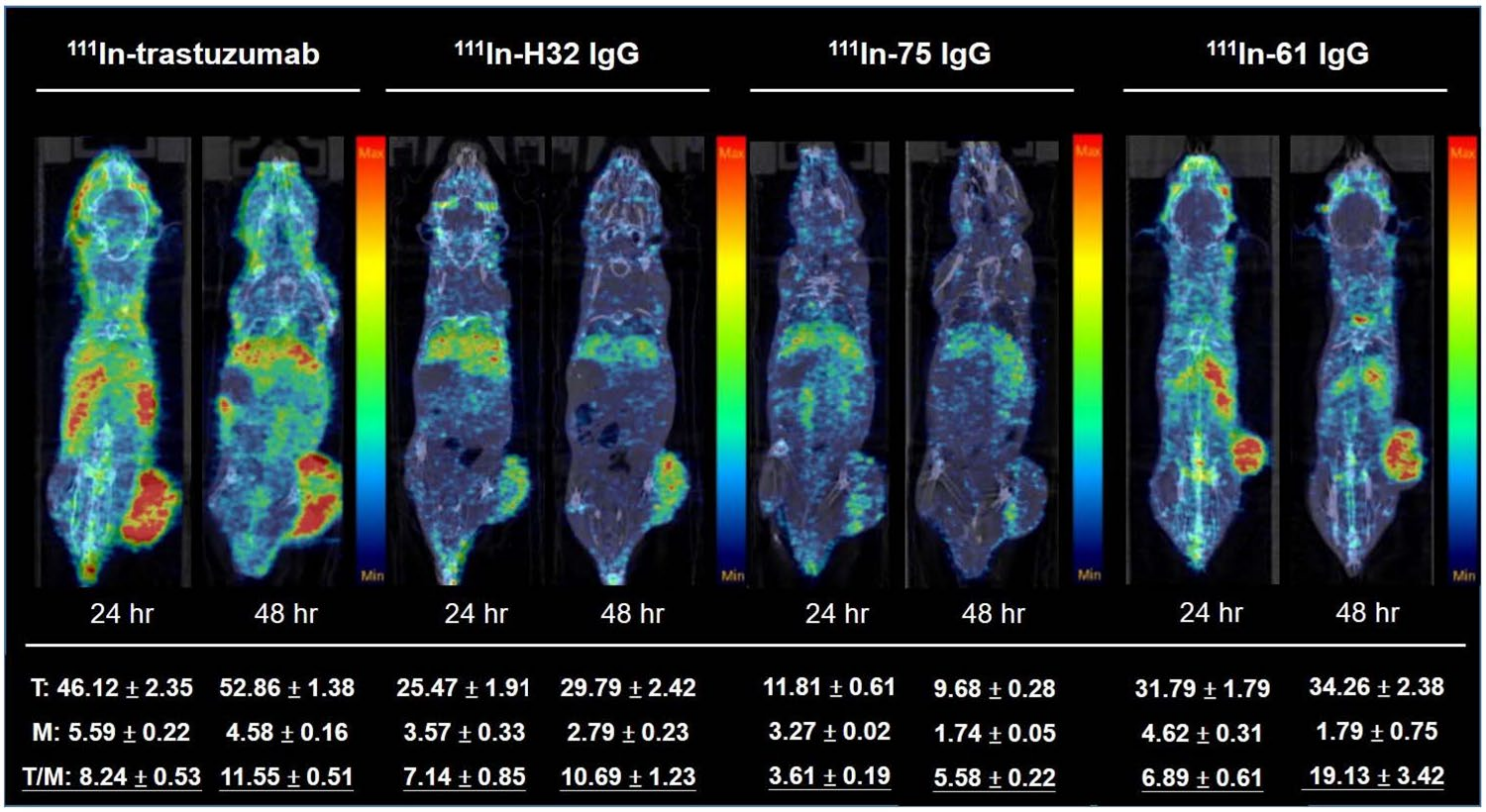
MicroSPECT/CT imaging of N87 xenograft-bearing mice injected with 18.5 MBq (0.1 mg/mouse) of [^111^In]labeled antibodies at 24 and 48 hr p.i.

Apparent liver uptake was noticed in each group because of phagocytic function of macrophage in liver (Fig. 5). The tumor uptake, derived from images, of mice administered with [^111^In]61 IgG, [^111^In]75 IgG, [^111^In]H32 IgG and [^111^In]trastutuzumab was 31.79 ± 1.79, 11.81 ± 0.61, 25.47 ± 1.91, and 46.12 ± 2.35 at 24 hr p.i., and was 34.26 ± 2.38, 9.68 ± 0.28, 29.79 ± 2.42, and 52.86 ± 1.38 at 48 hr p.i., respectively. The radioactivity of these antibodies accumulated in muscle declined over the experimental period, and this resulted in that the *T/M* increased with time in each group, even though [^111^In]75 IgG was washed out from the tumor, unlike the others. The *T/M* of the tumor-bearing mice at 48 hr after injection of [^111^In]61 IgG, [^111^In]75 IgG, [^111^In]H32 IgG and [^111^In]trastuzumab reached 19.13 ± 3.42, 5.58 ± 0.22, 10.69 ± 1.23, and 11.55 ± 0.51, respectively, demonstrating that these antibodies would be specifically retained in human gastric xenografts. We also found the radioactivity of [^111^In]labeled trastuzumab, H32 IgG, and 61 IgG significantly retained in BT474 human breast cancer xenografts as well as in N87 tumors (Figure S1b).

The difference in *T/M* of four groups between 24 and 48 hr p.i. were applied to determine the rate of internalization of each antibody-receptor complexes noninvasively. The relative uptake increment of [^111^In]H32 IgG-, [^111^In]61 IgG-, [^111^In]75 IgG- and [^111^In]trastuzumab-injected mice was 49.7%, 76.9%, 54.6%, and 40.2%, respectively, suggesting that H32 IgG was more resistant to internalization and stably stay at the cell surface, while 61 IgG preferred to enter the cell through endocytosis.

### Biodistribution studies

**Figure 6:**
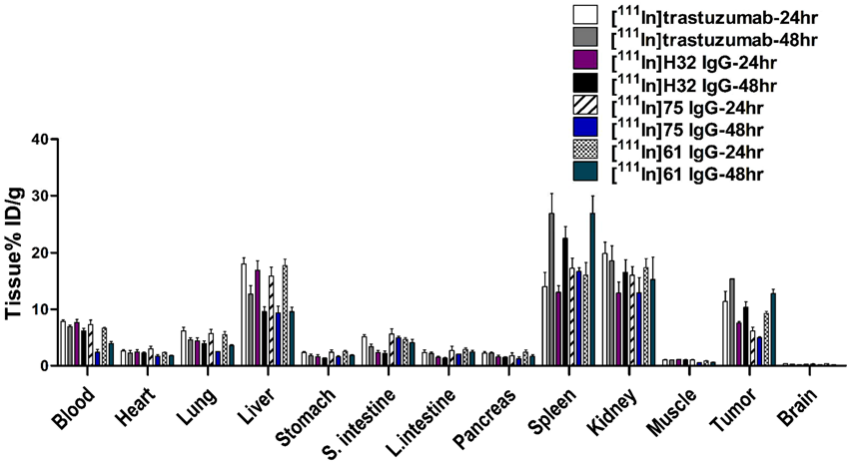
Biodistribution of [^111^In]labeled antibodies in N87 xenograft-bearing mice.

The liver uptake in each group was similar at 24 hr p.i., ranging from 15.90 ± 1.59 to 18.04 ± 1.05%ID/g, and then declined with time. The apparent radioactivity accumulation was also noticed in the spleen of the mice received an injection of [^111^In]labeled antibodies. The accumulations of [^111^In]H32 IgG, [^111^In]61 IgG, [^111^In]75 IgG and [^111^In]trastuzumab in the tumor were significantly higher than in most normal tissues at 24 hr after injection. Except for [^111^In]75 IgG, the maximum tumor uptake of [^111^In]H32 IgG, [^111^In]61 IgG, and [^111^In]trastuzumab all occurred at 48 hr p.i. and reached 10.32 ± 1.01, 12.75 ± 0.78, and 15.37 ± 0.01%ID/g, respectively. More importantly, these values were greater than those retained in liver (Fig. 6), indicating that these radiolabeled antibodies would be trapped in lesions over two days. The *T/M* attained the peak at 48 hr after injection in the four groups, and [^111^In]61 IgG owned the highest one. Derived from biodistribution studies, the relative uptake increment of [^111^In]H32 IgG-, [^111^In]61 IgG-, [^111^In]75 IgG- and [^111^In]trastutuzumab-injected mice was 38.50%, 90.02%, 62.68%, and 36.83%, respectively.

## Discussion

Anti-HER2 antibodies with different sequences would bind to different epitopes on the antigen and then lead to distinct biological effects. According to previous works, the binding sites of 61 IgG, 75 IgG, and H32 IgG was situated on the domain I of the ECD of HER2 and was different from that of pertuzumab (domain II) and trastuzumab (domain IV)^15^. The function or effect on the internalization kinetics of binding to domain I of HER2/ECD is not fully understood yet. Therefore, we would like to determine the feasibility of a multimodal noninvasive imaging platform to assess the *in vivo* biological properties and clinical potential of these self-developed anti-HER2 antibodies.

The internalization assays and western blotting indicated that H32 IgG would be internalized at the initial stage and then caused depletion of HER2 on the cell surface of N87 cells (Fig. 2b,c), suggesting that the depletion is the main reason for unsustained internalization of H32 IgG. Another possible explanation is the binding site-specificity of different antibodies, and this difference may result in different assembly of pinocytotic internalization mechanisms. Confocal assays confirmed the findings from *in vitro* internalization studies (Fig. 3). The pattern of confocal images of N87 cells treated with fluorescence-conjugated H32 IgG and trastuzumab at 37 °C was similar with that of cells incubated at 4 °C, implying that they did not preferentially enter into cells at 16 hr after incubation (Fig. 3). In contrast to H32 IgG and trastuzumab, the co-localization of 61/75 IgGs with intracellular lysosomes, were apparently evident, indicating that these two antibodies would be located in the cytoplasm and demonstrating their potential to play a role in anti-HER2 ADCs.

In microSPECT/CT imaging, we found that all radiolabeled antibodies were able to delineate tumor lesions (Fig. 5). Rudnick *et al*. reported that the cellular uptake level of antibodies inversely correlated with the affinity toward HER2 because C6.5-IgG is having the highest affinity (K_D_ = 2.7 × 10^–8^) had a lower uptake than trastuzumab did in SK-OV-3 human ovarian carcinoma tumor xenograft at 24 and 120 hrs post-injection^16^. Although the difference between H32 IgG (K_D_ = 2.7 × 10^−8^), 61 IgG (K_D_ = 2.7 × 10^−8^), and 75 IgG (K_D_ = 2.7 × 10^−8^) in affinity against HER2 was not obvious^15^, the inverse correlation between affinity and *in vivo* tumor uptake, either derived from images or obtained from biodistribution studies, was also noticed (Figs 5 and 6). Fractions of internalized HER2 receptors are recycled back to the cell membrane, assuming the radioactivity accumulation of “internalization-prone” antibody comes from both radioactivity inside and outside of cells and would be much higher than those are not transported into cells over time. In this study, we applied relative uptake increment to determine the changes in specific tumor uptake between early and late stage, and to some extent, this can be considered as an index for screening promising antibody component of the ADCs. [^111^In]61 IgG owned the highest relative uptake increment in both microSPECT imaging and biodistribution studies when compared with other antibodies, including trastuzumab (Figs 5 and 6). Although [^111^In]75 IgG had moderate *in vitro* internalization percentage and relative uptake increment, the retention of radioactivity in tumor decreased with time and only nearly a third part of that of [^111^In]61 IgG in the 48 hr p.i. imaging (Figs 2 and 5). We would assess whether human neonatal Fc receptor (FcRns), expressed within the endosomes and capable of transporting internalized antibodies back to the extracellular space, involves in this low-level *in vivo* uptake of [^111^In]75 IgG or not in the further experiments. In fact, this discrepancy between *in vitro* and *in vivo* findings highlighted the importance of noninvasive imaging platform, because it allows us to “predict” the biological properties of a specific antibody in the living body.

Regarding the noticeable liver uptake, concern about the liver toxicity may arise, especially when we aim to utilize these antibodies as a targeting component of ADCs. Sihver *et al*. noted that the radioactivity retention of [^125^I]labeled cetuximab in liver was significantly lower than that of radiometal-labeled one. However, the tradeoff is that [^125^I]cetuximab less accumulated in the tumor^17^. Decreased tumor and liver uptake were observed in microSPECT/CT images of N87 xenograft-bearing mice injected with [^123^I]61 IgG and [^123^I]trastuzumab at 12 and 36 hrs p.i. (Figure S2). High thyroid uptake, as well as rapid clearance from tumor highly suggested that they would be subjected to deiodination and were not feasible to monitor the internalization processes since the radioactivity showed in images cannot reflect the real distribution of parent antibody at later time points. Compared with directly radioiodinated on tyrosine residues of antibody, ^111^In was introduced to an antibody through a DTPA chelator, which is considered relatively stable. Sands *et al*. discovered that the activity in liver was primarily caused by the transchelation of In-111 to intracellular proteins in either hepatocytes or Kupffer cells^18^. The results from *in vivo* optical imaging echoed the previous findings because we did not notice the high-level liver signals in the mice injected with dye-conjugated 61 IgG and 75 IgG, while those injected with H32 IgG and trastuzumab showed significant liver retention. Taken together, this imaging platform, combining nuclear imaging with optical imaging, can eliminate the bias originated from single modality and can be used to evaluate the off-target effect with different “views.”

Antibody component of HER2-based ADCs is responsible for targeting and inducing receptor-medicated internalization to released cytotoxic agents inside the cancer cells rather than normal cells so that the safety profile of this ADC can be improved. The treatment response to T-DM1 has been proved in patients with HER2-overexpressed breast cancer, even in those has with HER2-positive trastuzumab-resistant breast cancer. Numerous preclinical or clinical trials using T-DM1 in other indications are being performed. This study demonstrated the potential of 61 IgG, one of the products from the previously developed GH2 library, by established imaging platform. To verify the reliability of this noninvasive technique to select optimal antibody for ADC, a study to compare the therapeutic efficacy of cytotoxin-conjugated 61 IgG and trastuzumab has been initiated recently.

## Conclusion

We have emphasized that limitation and inappropriateness of using NIRF imaging alone for assessment of tumor targeting and systemic biodistribution of anti-HER2 antibodies in this study. The microSPECT imaging of radiolabeled IgGs in combination with NIRF imaging may allow us a better understanding of the possible off-target effect of specific antibody. More importantly, We found that 61 IgG had superior/comparable *in vitro* cellular accumulation, internalization rate and *in vivo* xenograft uptake when compared with trastuzumab.

## Materials and Methods

### Preparation of the HER2-targeting NIRF imaging agent

The HER2-targeting near-infrared fluorescence (NIRF) imaging agents were generated by conjugating anti-HER2 antibodies (H32 IgG, 75 IgG, 61 IgG or trastuzumab) with DyLight680 succinimidyl ester (Pierce, Rockford, IL) as previously described^19^. Briefly, the antibody was mixed with the dye ester in bicarbonate buffer (pH 9.0) at a 6:1 molar ratio. After incubation for 1 hr at room temperature (r.t.), the DyLight680-anti-HER2 antibody conjugates, denoted as Dye-IgGs, was purified with a PD-10 desalting column (GE Healthcare) using phosphate buffered saline (PBS) as the mobile phase. The negative control was generated by conjugating isotype-matched control IgG with DyLight680 using the same protocol. The recovery rate of dye-conjugated 61, 75, H32 IgG and trastuzumab ranged from 60 to 75%. Their dye-to-antibody ratio equal to 2.

### Preparation of In-111 labeled antibodies

The anti-HER2 antibodies modified with diethylenetriaminepentaacetic acid (DTPA) were prepared based on the method published by Costantini *et al*. with minor modifications^20^. Briefly, a 5-fold molar excess of DTPA was added into a vial containing an anti-HER2 antibody, and the reaction mixture was reacted for 2 hr at 37 °C. After the reaction, the crude product was loaded onto a 30-kDa membrane column and then centrifuged at 5500 g for 10 mins. The residue was diluted with 400 µL of PBS and re-loaded to the 30-kDa membrane column for subsequent purification. For In-111 labeling, ^111^InCl_3_ was first incubated with 40 µL of sodium citrate buffer (0.1 M, pH = 5.0) for 10 min at r.t. DTPA-modified antibodies in PBS was added to the vial containing ^111^InCl_3,_ and then the reaction mixture was reacted for 50 mins at 37 °C. The reaction mixture was diluted to 400 μL and loaded onto a 30-kDa membrane column for purification. The radioactivity-containing column was centrifuged at 5500 g for 10 mins, and this purification procedure was repeated at least twice. The labeling efficiency and radiochemical purity of ^111^In-labeled antibody were determined by using a radio-thin layer chromatography (radio-TLC) scanner (AR2000; Bioscan). TLC was performed on an instant thin-layer chromatography plate coated with silica gel (ITLC-SG, Merck), using normal saline as the mobile phase. The radiochemical yield (decay non-corrected) of these antibodies ranged from 40% to 60% with a high radiochemical purity (>90%).

### Cell culture and animal model establishment

The N87 gastric cancer cell line (HER2-positive) was purchased from American Type Culture Collection (ATCC, Manassas, VA). Cells grow in RPMI 1640 medium (Invitrogen, Carlsbad, CA) supplemented with 10% fetal bovine serum (FBS) at 37 °C in a humidified atmosphere containing 5% CO_2_. The mouse experiments were performed by the Guidelines of the Academia Sinica Institutional Animal Care and Use Committee (IACUC #13-03-545). N87 cells (1 × 10^6^) in 100 µL of Matrigel and culture medium mixture (1:1, v/v) were implanted subcutaneously into the right flank of 6-week-old male NOD/SCID mice. When the tumor volume reached 100 mm^3^, the mice were selected for further experiments.

### HER2-binding affinity assays

The binding capacity of anti-HER2 antibodies was measured by ELISA method. Briefly, HER2 ECD was diluted to a final concentration of 300 ng/mL with PBS and plated on 96-well plates (100 μL/well) at 4 °C overnight. The coating solution was removed, and the remained protein-binding sites were blocked by incubation with 5% skim milk in PBST (0.05% (v/v) Tween 20) for 1 hr. Gradient concentration of anti-HER2 antibodies was added in triplicate. After 1-hr reaction, the plate was washed three times with 300 μL of PBST. Horseradish peroxidase-conjugated goat anti-human IgG Fc antibody was added to each well and incubated at r.t. for 1 hr. The plate was then washed three times with PBST and PBS. Finally, the 3,3′,5,5′-tetramethylbenzidine peroxidase substrate (horseradish peroxidase substrate) was added to each well for chromogenic reaction to develop for 5 mins. The HCl solution (1 N) was used to stop the reaction. The absorbance of each well was read at 450 nm with a plate reader (Victor 3 V 1420 Multilabel Counter, Perkin Elmer).

### Cellular uptake and internalization studies

Approximately 1 × 10^6^ cells were seeded in a 6-well plate and cultured in culture medium with 10% FBS (3 mL). The medium was aspirated at 24 hr after incubation, and the serum-free medium containing radiolabeled anti-HER2 antibodies (3 mL, 0.074 MBq/mL) was added to each well. At 4, 12, 24, and 48 hr post-incubation, the medium was removed, and the cells were washed twice with PBS (0.5 mL) to remove unbound radioactivity. Both medium and washing buffer were collected into a counting vial. The cells were treated with 0.5 mL of 0.5% trypsin for 5 mins to detach them from the plate. The cells were collected and resuspended in 1.5 mL of serum-containing medium to neutralize the activity of trypsin. A 40-µL of the sample was used to measure the number of cells in the cell suspension. The cellular uptake was expressed as the percentage of administered dose accumulated in one million cells (%AD/10^6^ cells).

For internalization assays, the procedure was according to the previously published method with some minor modifications^21^. Cells collected from cellular uptake assays were resuspended in a 1-mL solution of 200 nM sodium acetate and 500 nM sodium chloride (pH = 2.5) at 4 °C for 5 mins to remove the cell surface-bound radioactivity from cells. After the centrifugation at 8000 g for 10 mins, the cells were washed twice with PBS. Both supernatant and washing buffer were collected into a vial, and the cell pellets were added to another counting vial for measurement of radioactivity. The internalization rate was expressed as the percentage of initially bound activity.

### Endocytosis and transport of anti-HER2 antibodies

Anti-HER2 antibodies lysosomal trafficking was performed according to a published method^22^. Briefly, N87 cells in growth medium were seeded onto 8-well glass chamber slides (Millicell® EZ slides) at a density of 5 × 10^4^ cells per well. After 18 hr incubation, cells were incubated with Dye-anti-HER2 antibodies (10 nmol/L in PBS) for either 30 mins on ice or 16 hr at 37 °C. After washing twice with cold PBS, the cells were exposed to Fixation/Permeabilization solution (BD Biosciences) for 20 mins at 4 °C and washed twice with 1X Perm/Wash buffer (BD Biosciences). The lysosomal compartment was detected by a rabbit anti-human LAMP2 antibody (GeneTex, cat.GTX103214) and then visualized by Alexa Fluor 488 labeled goat anti-rabbit IgG secondary antibody (ThermoFisher). Cells were mounted with VECTASHIELD mounting medium containing DAPI (H-1200, VECTOR) and examined under a confocal microscope (Leica, TCS SP5).

### *In vivo* optical imaging

N87 tumor-bearing mice were randomly divided into five groups that intravenously injected with 0.5 nmol of Dye-H32 IgG, Dye-61 IgG, Dye-75 IgG, Dye-trastuzumab, and Dye-isotype control (as a negative control), respectively. At 24 hr after injection, the mice were imaged using a small-animal IVIS imaging system (Xenogen) with excitation and emission wavelengths of 675 and 720 nm, respectively. Fluorescence emission was normalized to photons per second per centimeter squared per steradian (p/s/cm^2^/sr).

### MicroSPECT/CT imaging

The microSPECT/CT images were acquired by the scanner at Chang Gung Memorial Hospital (nanoSPECT/CT, Mediso). The xenograft-bearing mice were randomly divided into four groups that were intravenously injected with 18.5 MBq (0.1 mg/mouse) of [^111^In]61 IgG, [^111^In]75 IgG, [^111^In]H32 IgG, and [^111^In]trastuzumab, respectively. Static imaging was carried out for around 30 mins at 24 and 48 hr after administration. Regions of interest (ROIs) were selected over the tumor and muscle, and the average values of the pixels within ROIs were corrected by subtracting the background radioactivity, which is measured in the remote areas away from the body. The specific tumor uptake was expressed as the tumor-to-muscle ratio (*T/M*) to eliminate the individual difference. The relative uptake increment represents the relative change between the *T/M* at 24 and 48 hr p.i. according to the following equation.

$${\rm{Relative}}\,{\rm{uptake}}\,{\rm{increment}}=\frac{T/{M}_{48}-T/{M}_{24}}{T/{M}_{24}}$$

where *T*/*M*_24_ and *T*/*M*_48_ was the *T/M* obtained from 24 hr and 48 hr microSPECT images, respectively.

### Assessment of biodistribution by tissue sampling

The mice in group 1, 2, 3 and 4 were received intravenous injection (i.v.) of 1.85 MBq (0.1 mg/mouse) of [^111^In]H32 IgG, [^111^In]61 IgG, [^111^In]75 IgG, and [^111^In]trastuzumab, respectively. The mice (n > 5) in each group were sacrificed by cervical dislocation at 24 and 48 hr p.i., and the tissues, including blood, heart, lung, liver, stomach, small intestine, large intestine, pancreas, spleen, muscle, kidney, bone, and tumor, were excised, cleaned, weighed, and counted by gamma counter (Cobra II, PerkinElmer Inc.). The radioactivity accumulated in tissues were expressed as the percentage of injected dose per gram (%ID/g).

### Statistical analysis

All data were expressed as the mean ± standard deviation (S.D.). The Student’s *t*-tests were applied for the comparison between groups. Values of *P* < 0.05 were considered as statistically significant.

### Ethical approval

This article does not contain any studies with human participants performed by any of authors. All applicable international, national, and institutional guidelines for the care of animals were followed. The animal experimental pertocol was approved by the institutional animal care and use committee of the China Medical University, Taichung, Taiwan (protocol No: 2017-180).

## Electronic supplementary material

Supplementary Information
