# Supplementary material for: Noninvasive assessment of characteristics of novel anti-HER2 antibodies by molecular imaging in a human gastric cancer xenograft-bearing mouse model
Source: Sci Rep. 2018 Sep 13;8:13735. doi: 10.1038/s41598-018-32094-x (PMC6137128; doi:10.1038/s41598-018-32094-x)
Supplement: Supplementary file 1 — Supplementary Information [file 41598_2018_32094_MOESM1_ESM.pdf]

## Supplementary Information

**Noninvasive assessment of characteristics of novel anti-HER2 antibodies by molecular imaging in a human gastric cancer xenograft-bearing mouse model**

Wei-Ying Kuo[1], Jia-Jia Lin[2], Hung-Ju Hsu[1], Hong-Sen Chen[1], *An-Suei Yang[1], and *Chun-Yi Wu[2,3]

[1]Genomics Research Center, Academia Sinica, Taipei, Taiwan
[2]Department of Biomedical Imaging and Radiological Science, China Medical University, Taichung, Taiwan
[3]Master Program for Biomedical Engineering, China Medical University, Taichung, Taiwan

* Correspondence should be addressed to: Chun-Yi Wu, Department of Biomedical Imaging and Radiological Science, China Medical University, Taichung, Taiwan 40402. Phone: +886-4-2205-3366 ext.7808. email: cywu@mail.cmu.edu.tw

**Supplementary Figure**

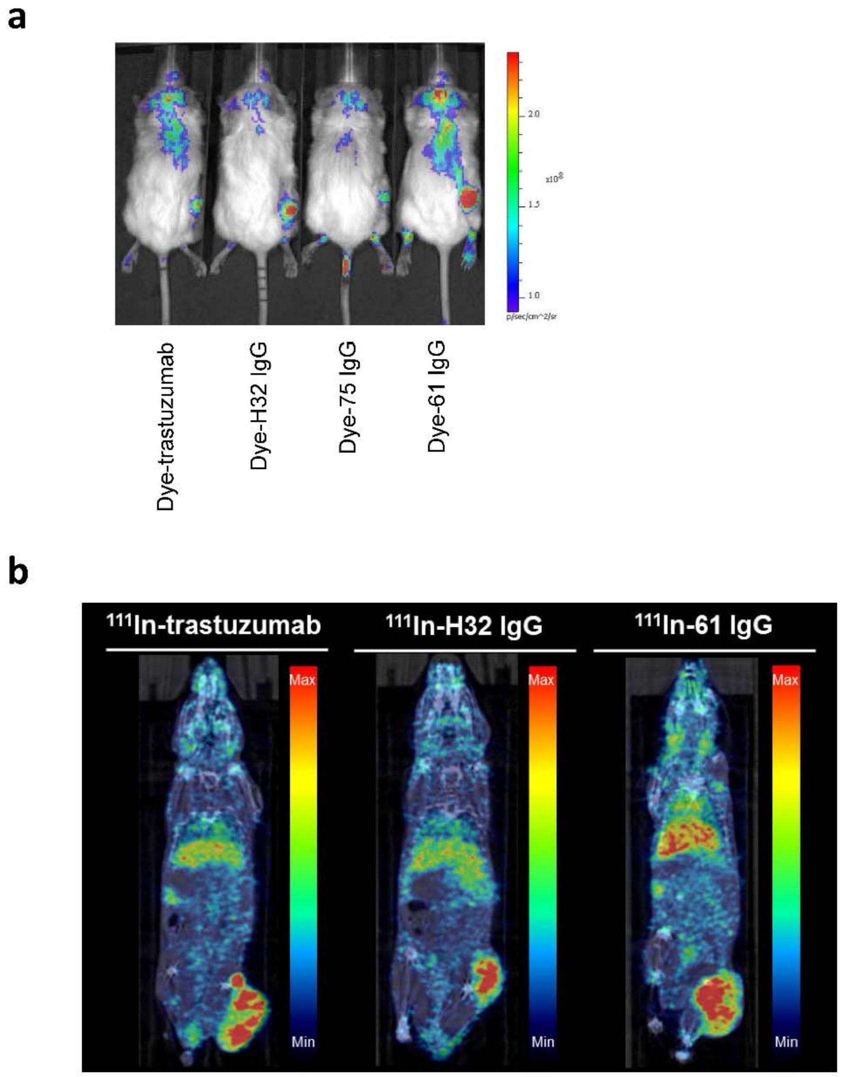


**Figure S1.** (a) *In vivo* NIRF imaging (b) MicroSPECT/CT imaging of BT474 xenograft-bearing mice injected with 18.5 MBq (0.1 mg/mouse) of [$^{111}$In]labeled antibodies at 24 hr p.i..

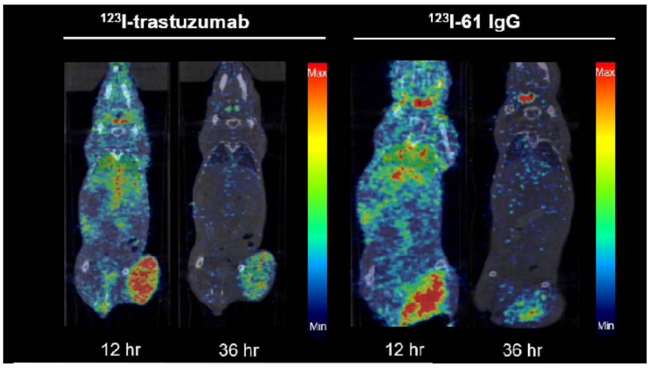


**Figure S2.** MicroSPECT/CT imaging of N87 xenograft-bearing mice injected with 18.5 MBq (0.1 mg/mouse) of [$^{123}$I]labeled trastuzumab and 61 IgG at 12 and 36 hr p.i..
